# Effects of Tea Polyphenols and Thermal Pretreatment on the Gel Properties and Storage Stability of Yogurt

**DOI:** 10.3390/foods15132365

**Published:** 2026-07-03

**Authors:** Xuhui Chen, Yaoyao Jiao, Longlin Wang, Yiheng Li, Chengrui Shi, Pengcheng Wen

**Affiliations:** 1 College of Food Science and Engineering, Gansu Agricultural University, Anning District, Lanzhou 730070, China; cxuhui1992@163.com (X.C.); wanglonglin5036@163.com (L.W.); yihenglixy@163.com (Y.L.); scr9809@163.com (C.S.); 2 School of Food Science and Technology, Dingxi Vocational and Technical College, Dingxi 730500, China; 15002556884@163.com

**Keywords:** tea polyphenols, yogurt, processing temperature, rheological properties, storage properties

## Abstract

This study investigated the effects of tea polyphenols (TPs) and thermal pretreatment on the gel properties and storage stability of set yogurt. Yogurt formulations were prepared with TP concentrations ranging from 0% to 0.3% and subjected to two heat treatments of 65 °C and 95 °C. Rheological measurements (storage modulus G′, loss modulus G″, and complex viscosity η*) showed that the addition of TPs improved gel viscoelastic properties, with greater effects under the 95 °C treatment. At a polyphenol level of 0.2%, the gel exhibited the highest stiffness and the most uniform microstructure, as confirmed by microscopy. Under 65 °C heating, increasing TP content led to reduced texture parameters, lower water-holding capacity, and greater syneresis, indicating an unstable gel. Conversely, the 95 °C-treated samples containing 0.2% TPs displayed optimal stability. During storage, all samples experienced declines in pH, water-holding capacity, and viscosity; however, the TP-enriched yogurts maintained significantly higher values for these parameters (*p* < 0.05) compared with controls, demonstrating superior structural retention. These results indicate that 0.2% TPs combined with 95 °C pretreatment improve yogurt gel properties and storage stability, with TPs showing potential as a functional ingredient for dairy applications.

## 1. Introduction

As consumers become more aware of health and wellness, many functional ingredients are gradually gaining popularity and being purchased by the public. Polyphenols and probiotics are already known to be effective functional foods. TPs, which possess polyhydroxy or galloyl groups, and their derivatives are recognized as potent antioxidants and exhibit anti-mutagenic and anti-cancer activities via pathways such as mutagen inactivation [1]. Accordingly, TPs are the generic designation of all phenolic compounds in tea, accounting for about 20–30% of the dry weight of tea leaves [2]. TPs are a heterogeneous family of phenolic compounds ubiquitously present in tea foliage, encompassing over 30 distinct phenolic constituents. Among these, catechins and their derivatives constitute the major bioactive fraction. Extensive phytochemical investigations have demonstrated that TPs possess a broad spectrum of physiological activities, including potent antioxidant, anti-aging, hypoglycemic, antibacterial, and enzyme-modulating properties [3]. The catechins are the main components of TPs, which account for 65–80% of the total amount of TPs. There are four main catechin compounds, including epicatechin (EC), epicatechin gallate (ECG), epigallocatechin gallate (EGCG), and epigallocatechin (EGC). The content of EGCG in green tea catechins was the highest, accounting for 50–60% of catechins. Interestingly, TPs are widely used in medicine, food, and agriculture due to their good antioxidant, anti-cancer, and cardiovascular disease prevention effects [4].

Phenolic compounds from various sources play an important role in regulating the quality of yogurt [5]. In view of the current market, more and more matcha ice cream, matcha yogurt, green tea cheese, and other foods based on milk formula are being introduced, mostly in the product development stage. TPs can bind to proteins, and the interaction is modulated by the polyphenols’ molecular weight, structural flexibility, and the abundance of hydroxyl groups. Among polyphenolic compounds, a particularly strong affinity for protein binding is observed with milk proteins [6,7]. It has been established that polyphenols with high molecular weight bind more strongly to proteins specifically [8]. EGCG, which possesses a high density of hydroxyl groups, interacts more strongly with proteins and therefore serves as a model phenolic compound for studying polyphenol–protein interactions. Consequently, modification of milk proteins by TPs alters protein structure and functional properties to varying extents [9]. In recent years, TP–milk protein interactions have been investigated more for the bioavailability of TPs and less for the functional properties of dairy products. Yogurt, as an important carrier of probiotics in the daily diet, occupies an important position in mass dietary consumption. The naturally stable structure of TPs is a key factor for their increased bioavailability during digestion, and the complexes with proteins prevent the oxidation of TPs as they pass through the gastrointestinal tract [10]. Dietary polyphenols are crucial for safeguarding the gastrointestinal tract against oxidative injury and may modulate cellular signaling pathways as well as influence the gut microbiota. Polyphenol–milk protein complexes have been employed as delivery carriers to achieve targeted release during digestion. The study evaluated pH and sensory attributes (texture, flavor, and color) of whole-milk yogurt stored at 4 °C on days 1, 7, and 35 with varying levels of olive bittersweet, a functional phenolic compound. The pH remained between 4.24 and 4.45 throughout storage, and the addition of olive bittersweet did not significantly affect any sensory property [11]. Du et al. [5] reported that polyphenol-enriched mulberry pomace extract reduced syneresis and improved texture in yogurt after fermentation. Najgebauer-Lejko et al. [12] reported that Green tea addition produced yogurts with a more consolidated gel structure and tighter interacting water than Pu-erh tea. TP–protein interactions are categorized into non-covalent and covalent interactions, which are the two most essential factors affecting the quality of TP-rich foods [13]. Early studies have shown that the modification of proteins by TPs occurs through the intermolecular formation of non-covalent bonds such as hydrogen bonding, hydrophobic interactions, ionic bonding, and van der Waals forces [14]. Additionally, the mechanism of covalent modification is considered to be that the irreversible covalent association between TPs and proteins arises from oxidative and nucleophilic addition processes of polyphenols [15]. The interaction of TPs with milk proteins is affected by some factors, including protein sequence and structure, chemical structure of polyphenols and their oxidation products, temperature, pH, and relative concentration of polyphenols and proteins. Hasni et al. [16] revealed that TP–milk protein interactions were strengthened with an increase in the number of hydroxyl groups (OH) in polyphenol compounds. Tea polyphenol binding altered the casein secondary structure, accompanied by an increase in the number of random coils and a decrease in α-helix and β-folding, leading to unfolding of the protein structure.

The exogenous phenolics are able to interact with milk protein and peptides and affect the metabolism of the lactic acid bacteria (LAB), which can be used in the production of functional yogurt before or after fermentation. It is generally believed that the interaction between phenolic substances and other factors in the system is closely related to the environment during the production of yogurt with polyphenols. Meantime, the effect of polyphenols on the physicochemical properties and functional properties of yogurt is inextricably linked to the interactions of dairy components.

To evaluate the effects of tea polyphenols (TPs) and thermal pretreatment on the gel properties and storage stability of yogurt, skim milk was subjected to thermal pretreatment at either 65 °C or 95 °C, followed by the addition of TPs at concentrations ranging from 0 to 0.3% before fermentation. The gel properties were comprehensively characterized by rheological analysis, texture profile analysis, microstructure observation, water-holding capacity, and syneresis measurements, all of which were also monitored during refrigerated storage to assess stability. The working hypothesis was that pretreatment at 95 °C would more effectively improve gel properties and stability than pretreatment at 65 °C, owing to the differential extent of protein denaturation and network formation induced by the two temperatures. The results suggest that the combination of TPs and high-temperature pretreatment enhances yogurt gel properties, indicating their potential as functional ingredients in dairy products.

## 2. Materials and Methods

### 2.1. Materials

TPs (containing 98% catechins, of which epigallocatechin gallate (EGCG) accounts for 50% of total catechins) were purchased from Cool Bioengineering Co., Ltd (Hefei, China). Fresh whole milk (with 3.03 ± 0.01% protein, 3.57 ± 0.13% fat, and 0.52 ± 0.22% ash) was obtained from Yili Dairy (Lanzhou, China). Fermenting agents (*L. bulgaricus* and *S. thermophilus*) were bought from Danisco (China) Co., Ltd. (Kunshan, China) and employed as described by the manufacturer. Fresh milk was packed in sterile high-density polyethylene plastic drums, then transported back to the laboratory at 4 °C within 1 h, and finally stored at −20 °C until use.

### 2.2. Preparation of Set-Type Yogurt with TPs

Fresh milk was defatted by centrifugation at ~15,000× *g* for 15 min at 4 °C. Skimmed milk was then supplemented with 0%, 0.1%, 0.2%, or 0.3% (*w*/*v*) TPs and stirred for 30 min. The samples were pasteurized at either 65 °C for 30 min or 95 °C for 5 min, cooled to 42 °C, and inoculated with 3% activated starter culture. Notably, the 65 °C/30 min condition represents a low-temperature long-time (LTLT) pasteurization protocol, which was deliberately chosen to minimize thermal denaturation of whey proteins and thereby preserve a greater proportion of native protein structure. The mixtures were stirred, aliquoted into 100 mL glasses, and fermented at 42 °C. pH was monitored every 30 min, and fermentation was stopped at pH 4.6. Fermentation time was recorded to capture acidification kinetics. All fermentation curves were performed in triplicate, where each replicate represents an independently prepared yogurt batch. The set yogurt was stored at 4 °C until analysis.

### 2.3. Rheological Characteristics

The rheological assay was performed by referring to the method of Zygmantaitė et al. with slight modifications [17]. Rheological measurements were performed using a rotational rheometer (MCR301, Anton Paar, Graz, Austria) equipped with a 50 mm diameter parallel plate geometry (PP50 probe). The gap was set at 1.0 mm. Approximately 2 mL of the yogurt sample was loaded onto the lower plate, excess sample was trimmed with a spatula, and a thin layer of silicone oil was applied around the geometry edge to prevent evaporation. The apparent viscosity was measured at a shear rate of 0.01–100 s^−1^. The storage modulus (G′), loss modulus (G″), and complex viscosity (η*) were measured by frequency sweep at 0.01–100 rad/s under a constant strain of 0.1% at 25 °C. All measurements were performed in triplicate, with each replicate representing an independently prepared yogurt batch.

### 2.4. Textural Properties

Texture properties were evaluated using a back extrusion test, following the method reported by Zhu et al. [18]. The test was performed on a TA.XT Plus texture analyzer (Stable Micro Systems, Godalming, UK) equipped with an A/BE-35 back-extrusion probe. Yogurt samples (150 mL) were measured at 25 °C. The test parameters were set as follows: pre-test speed 5.0 mm/s, test speed 2.0 mm/s, post-test speed 5.0 mm/s, and penetration depth 20 mm. Three independent measurements were performed for each sample.

### 2.5. Syneresis Analysis

Syneresis was measured based on the procedure previously described by Cardines et al. [19]. Briefly, 25 g of yogurt samples (sampling at the end of fermentation and fixed time at 0, 1, 2, 3, 4, 5, 6, 7 days during storage) were centrifuged using a refrigerated centrifuge (TGL-20, Xiangyi Centrifuge Instrument Co., Ltd., Changsha, China) at 4000× *g* for 20 min at 4 °C. The precipitate was removed, and the supernatant was weighed. The syneresis was calculated according to the following Equation (1):

(1) Syneresis (%) = W_1_/W_0_ × 100

where W_1_ is the weight of the supernatant, and W_0_ is the weight of the yogurt sample (25 g).

### 2.6. Confocal Laser Scanning Microscopy Analysis

Yogurt samples were placed on grooved glass slides and stained with one drop of 0.2% (*w*/*v*) fast green fluorescent dye for 2 min, and then rinsed with PBS to remove excess dye. The stained samples were covered with a coverslip and stored at 4 °C for 1 h. Then, the protein network structure was analyzed using confocal laser scanning microscopy (LSM800, Zeiss, Oberkochen, Germany) under an oil microscope, and the objective with an excitation wavelength of 633 nm. The microstructure of multiple regions of the yogurt was observed, and representative microscopic images were selected [20].

### 2.7. Physicochemical Properties of Yogurt During Storage

#### 2.7.1. Determination of pH and Viscosity

The viscosity was determined using a digital rotational viscometer (LVDV-1, Shanghai Fangrui Instrument Co., Ltd., Shanghai, China) at 4 °C. Samples were stirred for 40 s before measurement. The viscosity values were expressed in Pa·s and measured at 20 rpm with spindle No.4. The measurements were performed once per day for 7 consecutive days during storage at 4 °C. The pH was also measured using a pH meter at fixed time intervals for a total of 7 days.

#### 2.7.2. Water Holding Capacity Analysis

Water-holding capacity (WHC) was determined using a centrifugal procedure with slight modification [21]. Briefly, 25 g of yogurt samples (sampling at the end of fermentation and fixed time at 0, 1, 2, 3, 4, 5, 6, 7 days during storage) were centrifuged using a refrigerated centrifuge (TGL-20, Xiangyi Centrifuge Instrument Co., Ltd., Changsha, China) at 4000× *g* for 20 min at 4 °C. The supernatant was removed, and the precipitate was weighed. WHC was calculated according to the following Equation (2):

(2) WHC (%) = W_2_/W_0_ × 100

where W_2_ is the weight of the precipitate, and W_0_ is the weight of the yogurt sample (25 g).

It should be noted that WHC, as determined herein, refers to water retention after centrifugation, consistent with established practice in yogurt gel characterization.

#### 2.7.3. Color Determination

The color of yogurt samples was determined according to the methodology proposed by Popescu et al. and Li et al. [22,23]. Color determination was performed using a CR-410 colorimeter (Konica Minolta, Tokyo, Japan) with the CIELab system, calibrated against standard black and white plates before measurement. Luminance (L*), red-green component (a*), and yellow-blue component (b*) were recorded. The total color difference (ΔE) was calculated according to Equation (3):

(3) $$∆E*=\sqrt[{2}]{\left[{\left(L^{*}-L_{0}^{*}\right)}^{2}+{\left(a^{*}-a_{0}^{*}\right)}^{2}+{\left(b^{*}-b_{0}^{*}\right)}^{2}\right]}$$

where $L_{0}^{*}$ , $a_{0}^{*}$ and $b_{0}^{*}$ represent the initial color values of the yogurt samples ( with 0%, 0.1%, 0.2%, 0.3% TPs) on Day 0, while $L^{*}$ , $a^{*}$ and $b^{*}$ represent the measured color values at each storage time point (Days 1–7). Therefore, ΔE* reflects the total color difference change relative to Day 0 for different storage durations and is used to evaluate color stability during storage.

### 2.8. Statistical Analysis

All tests were repeated in triplicate, and each yogurt sample was analyzed three times. The obtained results were expressed as mean ± standard deviations (SDs). Separate one-way ANOVAs were performed for each heat treatment temperature (65 °C and 95 °C) to evaluate the effect of TP concentration on yogurt quality parameters. Analysis of variance for all tests was conducted by Duncan’s multiple range test (*p* < 0.05) using SPSS 22.0 software (IBM, New York, NY, USA).

## 3. Results and Discussion

### 3.1. Effects of TPs on pH of Protein Gel

**Figure 1:**
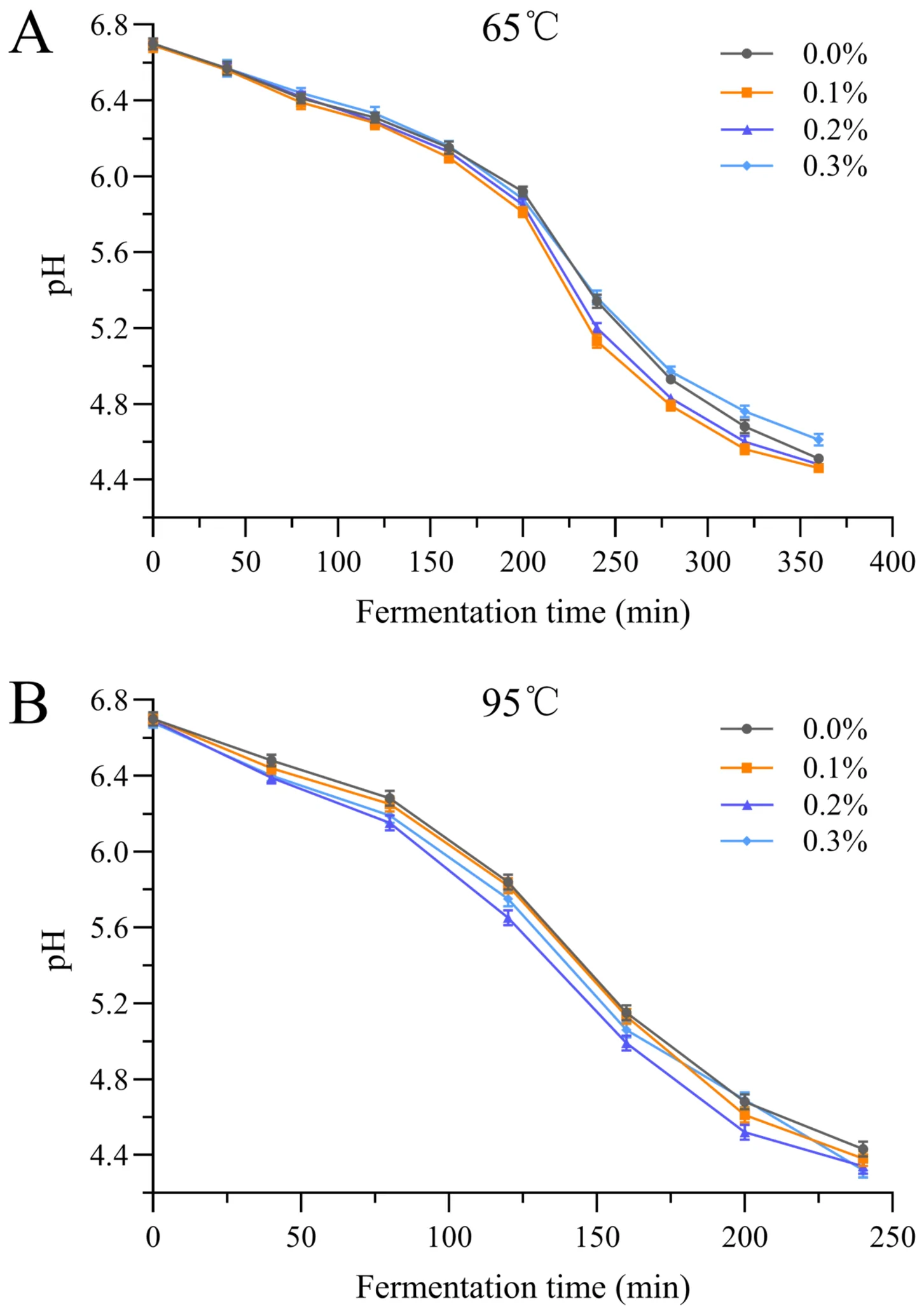
Fermentation time profiles of yogurt with various tea polyphenol levels under different thermal treatments. (**A**) Thermal treatment at 65 °C; (**B**) Thermal treatment at 95 °C. Different tea polyphenol concentrations are distinguished by line types and colors as shown in the legend.

Following heat treatment of milk and inoculation with a starter culture, fermentation was carried out at 42 °C. During this process, lactic acid bacteria (LAB) metabolize lactose in milk into lactic acid, thereby lowering the pH and inducing acidification of the milk [24]. The decline in pH gives rise to a reduction in the net negative charge of casein, which in turn weakens the electrostatic repulsion and spatial stability conferred by the κ-casein (κ-CN) charge layer on the surface of casein micelles. As a result, fragile colloidal networks are formed through hydrophobic interactions among adjacent casein micelles [17]. As depicted in Figure 1, milk protein samples and their corresponding TP–milk protein complexes displayed analogous trends in overall pH reduction with varying polyphenol addition levels. A pronounced acceleration in pH decline was observed for milk protein below pH 6.0, and the inclusion of TPs further promoted acidification during the fermentation of milk. At fermentation completion, the pH values of samples heat-treated at 65 °C were 4.51, 4.46, 4.48, and 4.61 for tea polyphenol concentrations of 0.0%, 0.1%, 0.2%, and 0.3%, respectively. By contrast, samples pretreated at 95 °C exhibited pH values of 4.43, 4.38, 4.34, and 4.32 at the same polyphenol levels. Moreover, the fermentation endpoint was reached at approximately 360 min following pretreatment at 65 °C, whereas only around 200 min was required for samples pretreated at 95 °C. This difference was mainly ascribed to the significantly shorter time span for pH to decrease from 6.0 to 5.0 under 95 °C treatment compared with 65 °C treatment. At 95 °C, extensive whey protein denaturation during pretreatment facilitated protein–catechin binding, thereby reducing the concentration of free catechins available to inhibit starter cultures and leading to monotonic pH decline. At 65 °C, limited protein denaturation left more free catechins in the gel matrix, which inhibited bacterial metabolism and caused a non-monotonic pH trend [25]. It should be noted that tea polyphenols may also directly affect the growth of microorganisms or enzymatic activity itself. Collectively, these results provide a scientific rationale for the common industrial practice of employing 95 °C for 5 min as a standard thermal sterilization regime in dairy manufacturing [17].

### 3.2. Effects of TPs on Rheological Characteristics of Protein Gel 

**Figure 2:**
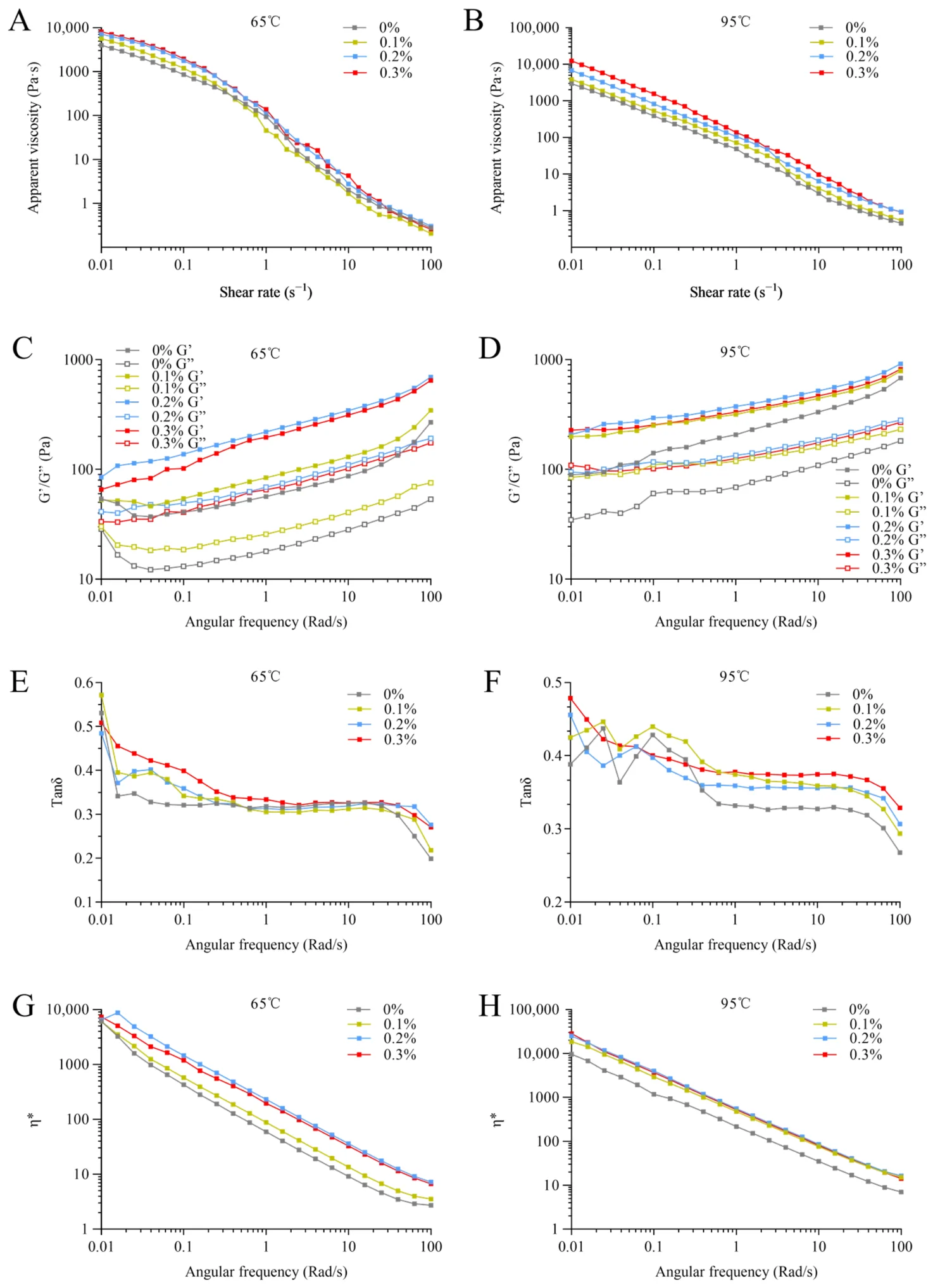
Rheological characteristic curves of yogurt incorporated with TPs under different thermal treatments. (**A**,**B**) Apparent viscosity of yogurt samples with TPs under 65 °C and 95 °C thermal treatments, respectively; (**C**,**D**) storage modulus (G′) and loss modulus (G″) of yogurt samples with TPs under 65 °C and 95 °C thermal treatments, respectively; (**E**,**F**) loss tangent (tan δ) of yogurt samples with TPs under 65 °C and 95 °C thermal treatments, respectively; (**G**,**H**) η* of yogurt samples with TPs under 65 °C and 95 °C thermal treatments, respectively.

Apparent viscosity represents a key functional attribute that directly influences consumer sensory acceptance, overall yogurt quality, and the structural stability of the fermented curd system, thus serving as an essential evaluation index in dairy product research [26]. The apparent viscosity of yogurt samples fermented with lactic acid bacteria from milk protein and TP–milk protein complexes is presented in Figure 2A,B. Over the shear rate range of 0.01 s^−1^ to 100 s^−1^ employed in the experiment, the apparent viscosity of all yogurt samples decreased sharply at low shear rates, whereas the reduction rate became milder at high shear rates, indicating typical shear-thinning behavior of non-Newtonian fluids [27]. Notably, the addition of TPs enhanced the viscoelastic properties under both heat treatments compared with the control. At 95 °C, apparent viscosity increased in a dose-dependent manner with TP concentration, indicating that TPs effectively reinforced the protein network. Overall, the apparent viscosity results confirmed that TPs addition increased the resistance to deformation of the yogurt gel.

Rheological characterization of yogurt gels provides critical insights into the effects of TPs on the structural integrity and mechanical performance of the protein network formed during fermentation. The evolution of storage modulus (G′) and loss modulus (G″) as a function of angular frequency (ω) for all yogurt samples is depicted in Figure 2C,D. Throughout the entire range of angular frequencies examined, all samples exhibited G′ values consistently higher than G″, indicating that the elastic modulus dominated the viscoelastic response [28]. Both G′ and G″ showed an upward trend with increasing angular frequency, reflecting frequency-dependent viscoelastic behavior characteristic of weak gel-like structures rather than ideal strong gels with frequency-independent modulus. These rheological profiles indicated that all yogurt samples possessed a weak gel-like structure. Notably, compared with the control and 0.1% TP treatment, the G′ and G″ values were higher at 0.2% and 0.3% TPs, with the 0.2% treatment yielding slightly higher values than the 0.3% treatment. This observation suggests that TP–milk protein interactions contributed to gel stiffness. Consistent with the aforementioned trends, the G′ and G″ values of yogurt samples subjected to 95 °C heat treatment were markedly higher than those treated at 65 °C. This phenomenon can be primarily attributed to the more extensive denaturation of whey proteins at the higher temperature (95 °C) [29]. Such thermal denaturation promoted stronger interactions between unfolded whey proteins and casein micelles, leading to the formation of denser and more extensive protein aggregates, thereby enhancing gel strength [30].

Loss tangent (Tanδ, G″/G′) is a critical parameter reflecting the viscoelastic behavior of gel networks [31]. Generally speaking, a higher Tanδ value signifies greater viscous contribution and correspondingly more fluid-like behavior, whereas a lower Tanδ indicates stronger elastic characteristics and a more solid-like nature. The Tanδ values of yogurt samples under different thermal treatments across the angular frequency range of 0.01 rad/s to 100 rad/s are presented in Figure 2E,F. Tanδ values fell below 1 over the entire range, consistent with the solid-like character expected of yogurt gels. During high-frequency scanning (1 rad/s to 100 rad/s), the Tanδ curves exhibited a slight downward tendency, suggesting that the elastic response became increasingly dominant under short-time external perturbations. By contrast, within the low-frequency region (0.1 rad/s to 1 rad/s), the Tanδ curves fluctuated more noticeably, accompanied by enhanced viscous characteristics. The fluctuations likely arise from multiple relaxation processes driven by heterogeneous protein–polyphenol interactions that shift the viscoelastic balance toward more viscous behavior at low frequencies. Comparing across treatments, TPs incorporation raised tan δ at low frequencies under both heat conditions, shifting the viscoelastic balance toward greater viscous character—an effect more pronounced at 65 °C. Across the entire frequency range, the 95 °C group gave consistently lower tan δ values (0.25–0.5) than the 65 °C group, indicating a more elastic-dominant gel network under the higher preheating temperature. This result further verified that the yogurt remained in a stable, solid-like state over the entire frequency scanning process and exhibited excellent resistance to shear deformation.

Compound viscosity (η*), often referred to as dynamic viscosity, characterizes the mechanical response of non-Newtonian fluids, which display both irreversible viscous deformation and reversible elastic deformation as a function of angular frequency [32]. Variations in η* as a function of angular frequency for yogurt samples fortified with TPs are illustrated in Figure 2G,H. Across the entire frequency range examined, η* exhibited a gradual decreasing trend for all yogurt formulations. Notably, yogurt samples containing different concentrations of TPs displayed higher η* values relative to the control group. This phenomenon can be explained by the increased viscoelasticity of the yogurt matrix, which requires overcoming molecular entanglement and internal friction among cross-linked protein chains during structural displacement [33]. At low angular frequencies, the non-covalent and partial covalent interactions formed between TPs and milk proteins further promoted intermolecular entanglement, thereby impeding molecular flow and elevating the overall complex viscosity. Meanwhile, yogurt samples subjected to 95 °C thermal treatment exhibited higher η* than those treated at 65 °C, which was mainly attributed to the enhanced denaturation of whey proteins and strengthened protein–protein interactions at the higher temperature [34]. Taken together, these results confirmed that the molecular interactions between TPs and milk proteins effectively contributed to the elevation of complex viscosity in the final yogurt products.

### 3.3. Effects of TPs on Textural Properties of Protein Gel

**Figure 3:**
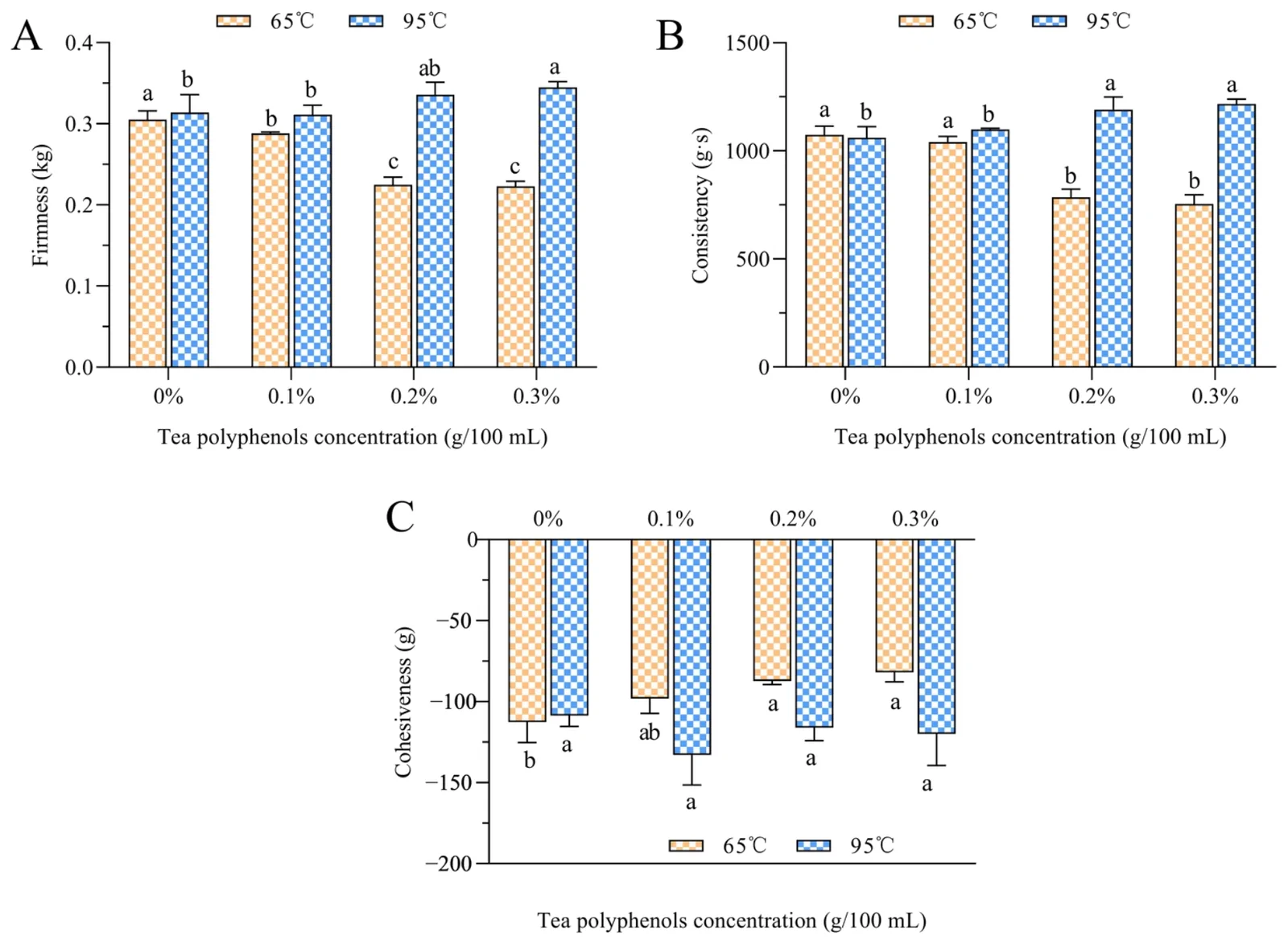
Texture properties of yogurt incorporated with TPs under different thermal treatments. (**A**) Firmness; (**B**) Consistency; (**C**) Cohesiveness. Different lowercase letters (a, b, c) indicate significant differences among treatment groups within the same thermal treatment (*p* < 0.05).

Textural properties serve as a vital indicator of yogurt quality and strongly influence consumer sensory perception and overall acceptance [35]. Firmness reflects the instantaneous force required to disrupt the gel network structure of set yogurt under external compression [36]. The effects of TPs on the firmness of yogurt samples are presented in Figure 3A. Compared with the control group, yogurt samples pretreated at 65 °C exhibited a significant decrease in firmness (*p* < 0.05) with increasing TP concentrations. Conversely, at a TP concentration of 0.3%, samples pretreated at 95 °C showed higher firmness relative to the control. These divergent trends under the two thermal conditions may be attributed to the distinct influences of different thermal treatment temperatures on the interaction between TPs and milk proteins [37].

As illustrated in Figure 3B, yogurt fortified with TPs displayed comparable variation tendencies in consistency following pretreatment at 65 °C and 95 °C. Under 65 °C pretreatment, consistency was significantly reduced (*p* < 0.05) at TP concentrations of 0.2% and 0.3% relative to 0% and 0.1%, with no significant difference observed between these two higher levels. In contrast, upon 95 °C pretreatment, consistency was significantly elevated (*p* < 0.05) at 0.2% and 0.3% TPs compared with 0% and 0.1% groups. Moreover, no significant difference in consistency was detected between the two thermal treatments in the absence of TPs, whereas yogurt samples containing 0.2% and 0.3% TPs exhibited markedly higher consistency at 95 °C than at 65 °C. The results are consistent with the aforementioned observations regarding apparent viscosity.

Cohesiveness reflects the deformability of a gel system prior to structural rupture, and is closely associated with the internal mechanical strength of the gel network [38]. As presented in Figure 3C, the influence of TPs on the cohesiveness of yogurt is displayed, with negative values merely representing the directional nature of the applied force. The results indicated that TP supplementation exerted similar regulatory effects on the cohesiveness of yogurt samples under both thermal conditions. As discussed previously, the gradual decrease in pH during yogurt fermentation led to continuous post-acidification. Under 95 °C pretreatment, this process contributed to improved consistency by reinforcing protein–protein interactions and facilitating the formation of a compact yogurt gel structure [39]. In addition, the presence of TPs enhanced the compactness of protein aggregates, thereby promoting the assembly of a more homogeneous and stable gel network through cross-linking with protein molecules.

### 3.4. Effects of TPs on Syneresis of Protein Gel

**Figure 4:**
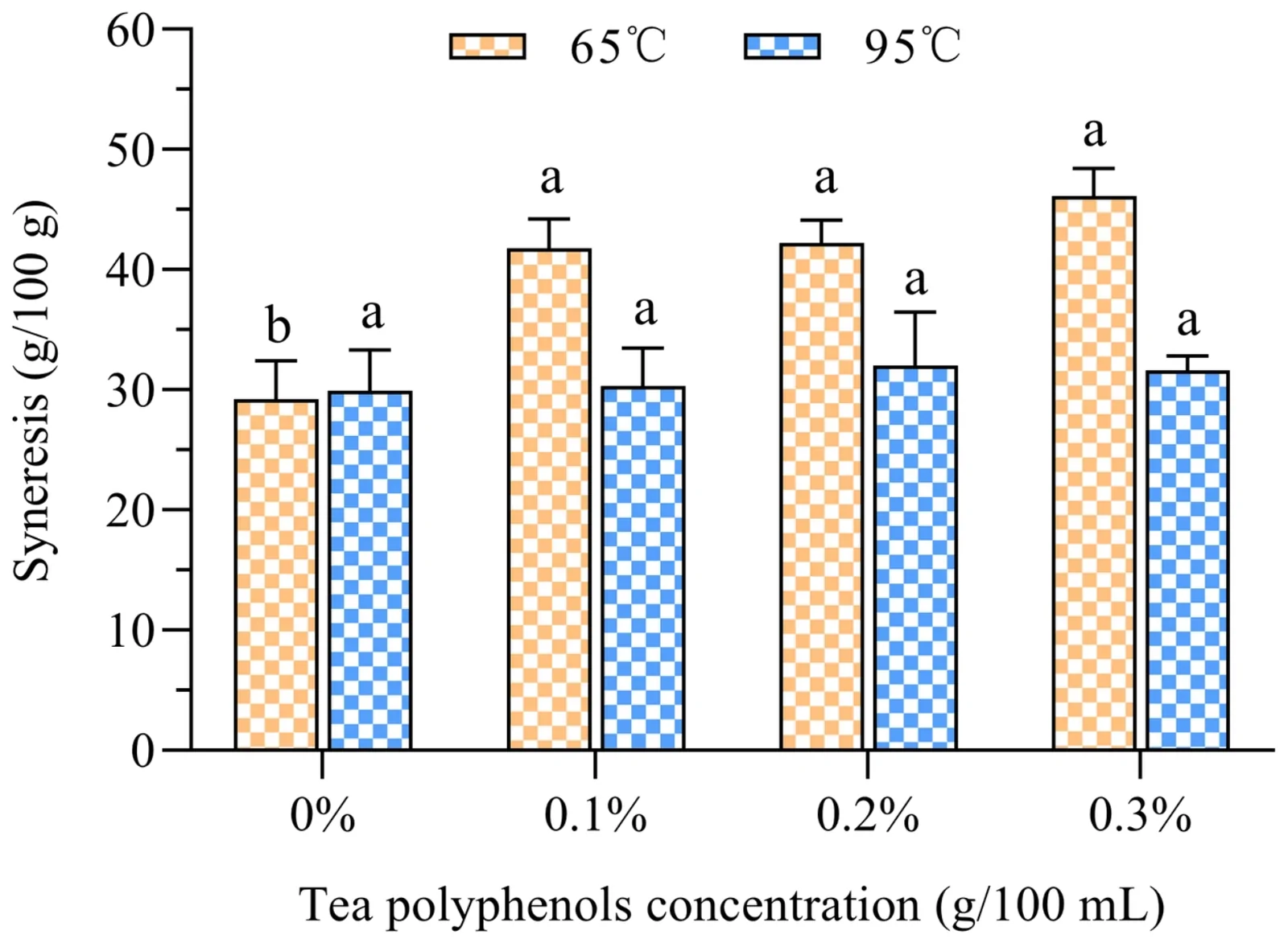
Syneresis analysis of yogurt incorporated with TPs under different thermal treatments. Different lowercase letters (a, b) indicate significant differences among treatment groups within the same thermal treatment (*p* < 0.05).

Syneresis is an inherent physical characteristic of set yogurt, generally originating from an unstable protein gel network and its insufficient water-holding capacity to retain whey [40]. Figure 4 illustrates the influence of TPs on syneresis after fermentation is complete. At 65 °C, syneresis rose markedly upon TPs addition (29.21 g/100 g in the control vs. 41.77–46.10 g/100 g with TPs, *p* < 0.05), with no further increase across the three dosage levels. Under the milder preheating condition, whey proteins remained largely in their native state, leaving a greater fraction of free catechins that weakened the casein network and promoted serum expulsion [41]. In the 95 °C pretreatment group, syneresis did not vary with TP concentration (*p* > 0.05), with all values fluctuating narrowly between 29.94 g/100 g and 32.34 g/100 g. By contrast, syneresis in the 95 °C group remained low and stable across TP levels, consistent with values considered acceptable for yogurt [42]. Under this condition, extensive whey protein denaturation promoted protein–TP binding, incorporating catechins into the gel network. TPs might form covalent modifications with thermally denatured whey proteins, further consolidating the gel network microstructure and alleviating whey exudation [43], thereby limiting whey separation and yielding consistently lower syneresis than the 65 °C group [41].

### 3.5. Microstructure of TPs-Protein Gel Complexes

**Figure 5:**
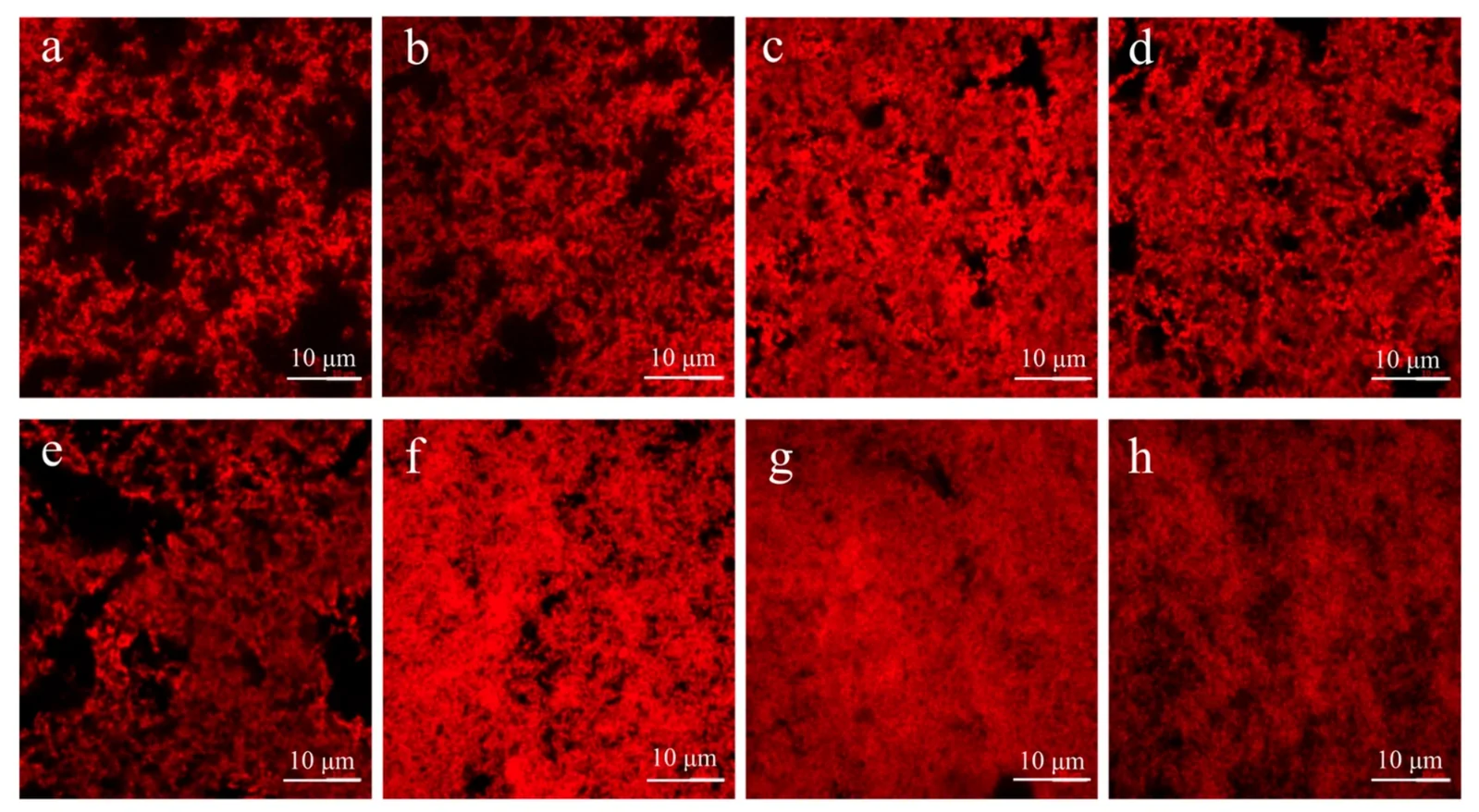
Confocal laser scanning microscopy (CLSM) images of yogurt samples supplemented with 0% (**a**,**e**), 0.1% (**b**,**f**), 0.2% (**c**,**g**), and 0.3% (**d**,**h**) tea polyphenols (TPs). Panels (**a**–**d**) represent samples pretreated at 65 °C, whereas panels (**e**–**h**) correspond to those pretreated at 95 °C.

All yogurt samples displayed a relatively uniform yet porous gel network structure as visualized by CLSM (Figure 5). In these micrographs, the red regions represented the protein-based gel network, and black areas corresponded to the aqueous phase containing low concentrations of whey proteins. As observed in Figure 5a–d, samples subjected to 65 °C pretreatment exhibited a gradually denser protein network accompanied by a reduction in the whey-rich phase with increasing TP levels. Similarly, under 95 °C pretreatment, yogurt samples fortified with TPs (Figure 5e–h) formed a markedly denser gel network compared with the TP-free control (Figure 5e). These observations confirmed that molecular interactions between TPs and milk proteins favored the development of a compact protein network with reduced free zones. Similar results were proposed in the studies of Du et al. [20] and Wu et al. [44]. Furthermore, previous studies have reported that polyphenols present in green coffee and green tea are capable of interacting with casein micelles in yogurt systems. Moreover, such protein–polyphenol interactions reinforce the casein network and stabilize the gel structure of yogurt by improving consistency and mitigating syneresis at optimal concentrations [45]. Notably, the gel network appeared most compact and densely structured at 0.2% TPs (Figure 5c,g), consistent with the trend observed in the rheological results. Comparative analysis between the two thermal treatments revealed that the gel structure formed at 65 °C was dominated by discrete protein particles, and such a particulate network tended to be unstable and susceptible to structural breakdown under external force [17,46]. These CLSM observations provide a reasonable explanation for the differences in rheological and textural properties of TP-fortified yogurt. The particulate network formed at 65 °C may entrap partial whey proteins under oscillatory shear, thereby leading to a moderate improvement in rheological behavior. Nevertheless, the overall gel structure remained relatively weak and unstable, since incompletely denatured whey proteins failed to fully occupy the interstitial voids [47]. In contrast, samples treated at 95 °C with TPs contained fewer protein particles and developed a more homogeneous and compact gel network. This effect can be attributed to the extensive denaturation of whey proteins at 95 °C, which facilitated their association with casein micelles mainly through disulfide bonds, thereby filling structural gaps and reinforcing the gel network [48]. Accordingly, these microscopic findings further support the observed improvements in the textural and rheological properties of yogurt.

### 3.6. Analysis of Physicochemical Properties of Yogurt During Storage

#### 3.6.1. pH Changes in Yogurt with TPs During Storage

**Figure 6:**
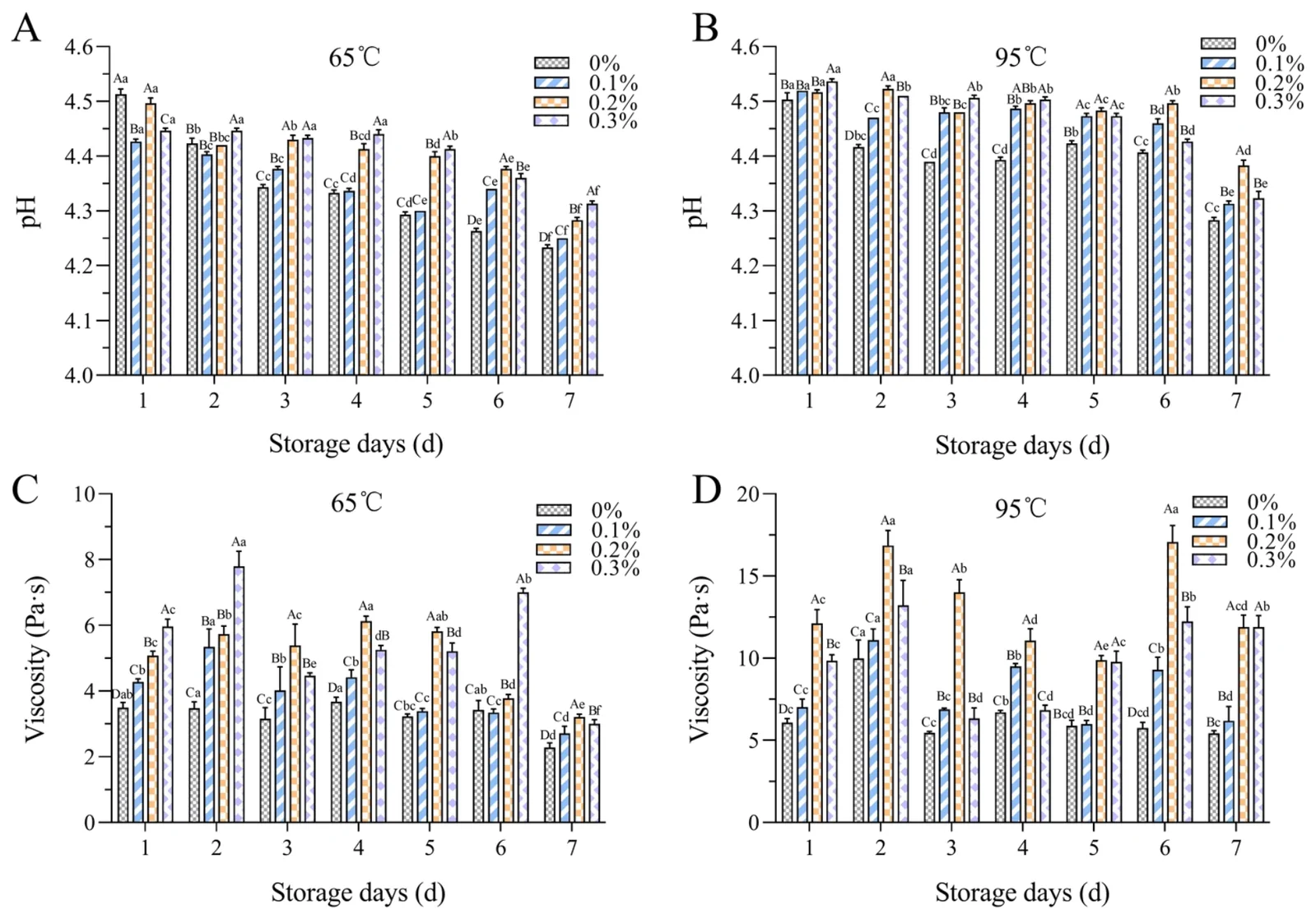
Changes in pH (**A**,**B**) and viscosity (**C**,**D**) of yogurt samples supplemented with tea polyphenols. Figure captions, uppercase letters denote significant differences among treatment groups within the same storage day, and lowercase letters denote significant differences among storage days within the same treatment group (*p* < 0.05).

The pH of all yogurt samples declined during 7 days of cold storage (Figure 6A,B). All samples started below pH 4.6 (the casein isoelectric point), consistent with a formed gel network [45]. TPs incorporation mitigated post-acidification. The extent of pH decline during storage varied with both TP concentration and heat pretreatment temperature. For yogurt subjected to 65 °C pretreatment, pH declined most sharply in the control and 0.1% TP groups; this decline was attenuated at 0.2% and 0.3% TPs. Meanwhile, under 95 °C thermal pretreatment, all TP-fortified yogurt samples exhibited slower pH decline relative to the control group. This phenomenon indicated that interactions between TPs and milk proteins effectively restrained the continuous drop in pH during cold storage, thereby extending the shelf stability of yogurt. In addition, this may be related to the slowing of bacterial growth during post-acidification. Across both heat treatments, the 95 °C group showed a slower and more stable pH decline than the 65 °C group. The stabilizing effect was most pronounced at 0.2% TPs under 95 °C, indicating that extensive whey protein denaturation reinforced the suppressive effect of TPs on post-acidification.

#### 3.6.2. The Viscosity Changes in Yogurt with TPs During Storage

As presented in Figure 6C,D, the viscosity of all yogurt samples displayed an initial rising followed by a subsequent declining trend throughout the 7-day cold storage. For samples subjected to 65 °C pretreatment, viscosity values ranged from 2.22 Pa·s to 7.80 Pa·s, while those under 95 °C treatment varied between 5.43 Pa·s and 17.07 Pa·s. Overall, yogurt pretreated at 95 °C maintained consistently higher viscosity in comparison with the 65 °C group. This discrepancy could be explained by the combined effects of thermal pretreatment and post-fermentation acid production by lactic acid bacteria. Compared with 65 °C treatment, the higher temperature of 95 °C induced greater denaturation of whey proteins, which further interacted with casein micelles to construct a denser gel network within yogurt. Furthermore, all TP-fortified samples at both thermal levels possessed higher viscosity than the corresponding control groups, and remarkable variations in viscosity occurred along with storage progression (*p* < 0.05). Overall, the decrease in viscosity during storage parallels the reduction in WHC and the increase in syneresis, reflecting the gradual weakening of the gel network. Samples containing 0.2% TPs exhibited the highest viscosity retention at 95 °C, which is consistent with their denser microstructure and superior WHC, indicating that a stronger protein–polyphenol network is better able to resist structural degradation during storage.

#### 3.6.3. Water Holding Capacity Changes in Yogurt with TPs During Storage

**Figure 7:**
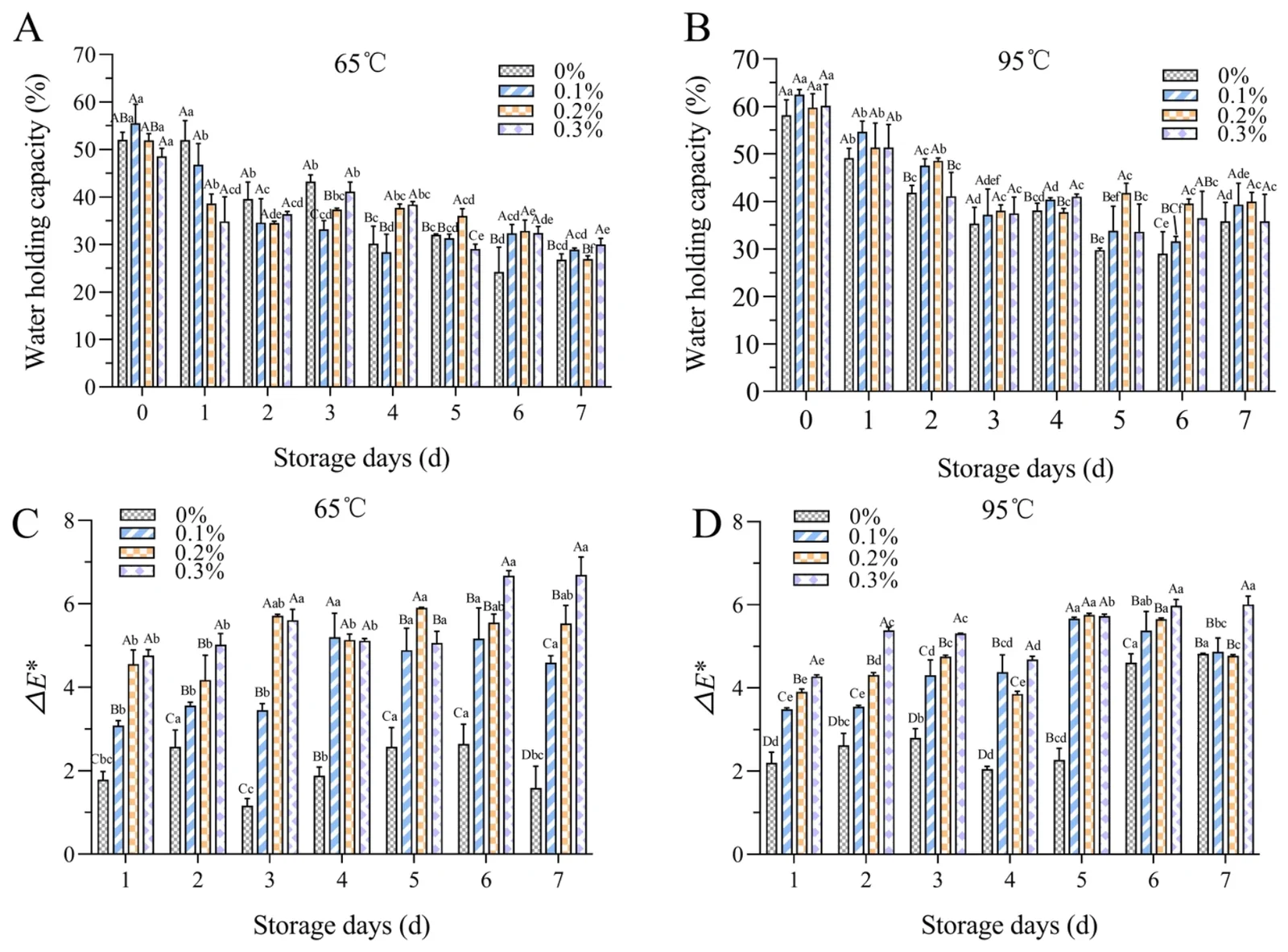
Changes in water holding capacity (**A**,**B**) and total color difference (**C**,**D**) of yogurt samples supplemented with tea polyphenols. Figure captions, uppercase letters denote significant differences among treatment groups within the same storage day, and lowercase letters denote significant differences among storage days within the same treatment group (*p* < 0.05).

Water holding capacity (WHC) is a crucial property determining the product yield, sensory quality, storage stability, and textural characteristics of yogurt [20]. As illustrated in Figure 7A,B, the WHC of all yogurt samples presented a continuous declining trend throughout the 7-day cold storage, featuring a rapid reduction in the early storage stage followed by a gradual slowdown in the later period. All yogurts in the present study were within this acceptable range. Such variations in WHC may be related to the exposure of hydrophobic groups on protein molecules during cold storage, which could strengthen hydrophobic interactions and reduce the water-binding ability of the gel matrix. Notably, yogurt samples pretreated at 95 °C exhibited overall higher WHC than those under 65 °C treatment. This phenomenon likely reflected more extensive whey protein denaturation at 95 °C, which reinforced the gel network and reduced whey separation. Moreover, under both 65 °C and 95 °C pretreatment conditions, TP-supplemented yogurt displayed significantly greater WHC than the control groups during storage (*p* < 0.05). These results were consistent with the findings reported by Du et al. [20] and could be explained by the strong binding affinity between TPs and milk proteins. The covalent and non-covalent intermolecular interactions formed between TPs and milk proteins promoted the assembly of stable protein complexes. Meanwhile, reinforced hydrophobic interactions and newly formed hydrogen bonds further improved the overall integrity and water-retention capacity of the yogurt gel network [49]. This mechanism, however, remains to be verified by direct structural evidence.

#### 3.6.4. Color Changes in Yogurt with TPs During Storage

Color variations in yogurt samples over the 7-day cold storage are displayed in Figure 7C,D. Overall, the ΔE* values for yogurt containing TPs were significantly higher than those of the control group at two heat treatment temperatures (*p* < 0.05). At 65 °C, the ΔE* values of all TP supplement groups generally showed an upward trend as storage time increased; the 0.2% and 0.3% groups reached 5.53 ± 0.36% and 6.69 ± 0.35% toward the end of storage, respectively, while the 0.1% TP supplement group remained lower (4.59 ± 0.14%). At 95 °C, the ΔE* values for all treatment groups also increased with prolonged storage time, but the rate of increase was more gradual. The values in the 0.2% and 0.3% TP supplement groups reached 5.17 ± 0.57% and 6.01 ± 0.16%, respectively. The ΔE* values increased with rising tea polyphenol concentration, likely due to the inherent color of the polyphenols.

## 4. Conclusions

This study examined the effects of TPs and thermal pretreatment on yogurt gel formation and stability. The 95 °C and 65 °C pretreatments affected protein–polyphenol interactions differently, which in turn changed rheological performance, texture, microstructure, and storage stability. Adding the right amount of TPs strengthened the protein gel network, and the 95 °C pretreatment further improved gel mechanical properties. At 0.2% TPs, the gel reached the highest stiffness and showed the most uniform microstructure, which CLSM confirmed. In contrast, as TPs increased, the 65 °C pretreatment resulted in a weaker gel structure, as evidenced by poorer texture, lower WHC, and more pronounced syneresis. During storage, all samples showed gradual drops in pH, viscosity, and water holding capacity. Still, TP-fortified yogurts kept better physicochemical properties than the control (*p* < 0.05), which points to better storage stability. In sum, adding 0.2% TPs with 95 °C pretreatment improved yogurt gel properties and storage stability. TPs show potential as a functional ingredient, but more work is needed to test them against commercial stabilizers. TPs can also cause bitterness and browning, which may hurt sensory acceptance. Future studies using FTIR, DSC, or similar techniques would help pin down how TPs interact with milk proteins.

## Data Availability

The original research findings presented in this article have been included in the paper. If you have any further questions, please contact the corresponding author.
